# Application of machine learning for detecting high fall risk in middle-aged workers using video-based analysis of the first 3 steps

**DOI:** 10.1093/joccuh/uiae075

**Published:** 2025-01-10

**Authors:** Naoki Sakane, Ken Yamauchi, Ippei Kutsuna, Akiko Suganuma, Masayuki Domichi, Kei Hirano, Kengo Wada, Masashi Ishimaru, Mitsuharu Hosokawa, Yosuke Izawa, Yoshihiro Matsumura, Junichi Hozumi

**Affiliations:** Division of Preventive Medicine, Clinical Research Institute, National Hospital Organization Kyoto Medical Center, 1-1 Mukaihata-cho, Fukakusa, Fushimi-ku, Kyoto 612-8555, Japan; Institute of Physical Education, Keio University, 4-1-1 Hiyoshi, Kohoku-ku, Yokohama, Kanagawa 223-8521, Japan; Division of Preventive Medicine, Clinical Research Institute, National Hospital Organization Kyoto Medical Center, 1-1 Mukaihata-cho, Fukakusa, Fushimi-ku, Kyoto 612-8555, Japan; Division of Preventive Medicine, Clinical Research Institute, National Hospital Organization Kyoto Medical Center, 1-1 Mukaihata-cho, Fukakusa, Fushimi-ku, Kyoto 612-8555, Japan; Division of Preventive Medicine, Clinical Research Institute, National Hospital Organization Kyoto Medical Center, 1-1 Mukaihata-cho, Fukakusa, Fushimi-ku, Kyoto 612-8555, Japan; Department of Electric Works Company/Engineering Division, Panasonic Corporation,1006, Kadoma, Kadoma City, Osaka 571-8501, Japan; Department of Electric Works Company/Engineering Division, Panasonic Corporation,1006, Kadoma, Kadoma City, Osaka 571-8501, Japan; Department of Electric Works Company/Engineering Division, Panasonic Corporation,1006, Kadoma, Kadoma City, Osaka 571-8501, Japan; Department of Electric Works Company/Engineering Division, Panasonic Corporation,1006, Kadoma, Kadoma City, Osaka 571-8501, Japan; Department of Electric Works Company/Engineering Division, Panasonic Corporation,1006, Kadoma, Kadoma City, Osaka 571-8501, Japan; Department of Electric Works Company/Engineering Division, Panasonic Corporation,1006, Kadoma, Kadoma City, Osaka 571-8501, Japan; Department of Electric Works Company/Engineering Division, Panasonic Corporation,1006, Kadoma, Kadoma City, Osaka 571-8501, Japan

**Keywords:** gait analysis, accidental falls, falls, machine learning, risk assessment, workplace, middle age

## Abstract

Objectives: Falls are among the most prevalent workplace accidents, necessitating thorough screening for susceptibility to falls and customization of individualized fall prevention programs. The aim of this study was to develop and validate a high fall risk prediction model using machine learning (ML) and video-based first 3 steps in middle-aged workers.

Methods: Participants to provide training data (*n* = 190, mean [SD] age = 54.5 [7.7] years, 48.9% male) and validation data (*n* = 28, age = 52.3 [6.0] years, 53.6% male) were enrolled in this study. Pose estimation was performed using a marker-free deep pose estimation method called MediaPipe Pose. The first 3 steps, including the movements of the arms, legs, trunk, and pelvis, were recorded using an RGB camera, and the gait features were identified. Using these gait features and fall histories, a stratified *k*-fold cross-validation method was used to ensure balanced training and test data, and the area under the curve (AUC) and 95% CI were calculated.

Results: Of 77 gait features in the first 3 steps, we found 3 gait features in men with an AUC of 0.909 (95% CI, 0.879-0.939) for fall risk, indicating an “excellent” (0.9-1.0) classification, whereas we determined 5 gait features in women with an AUC of 0.670 (95% CI, 0.621-0.719), indicating a “sufficient” (0.6-0.7) classification.

Conclusions: These findings suggest that fall risk prediction can be developed based on ML and the first 3 steps in men; however, the accuracy was only “sufficient” in women. Further development of the formula for women is required to improve its accuracy in the middle-aged working population.

## Teaser

Key points: We developed a machine learning–based fall risk prediction model using 3 gait features, achieving “excellent” performance with an AUC of 0.909 in men. However, using 5 gait features achieved only “sufficient” performance in women, with an AUC of 0.670.

## 1. Introduction

The obligation for employment until the age of 70 has been imposed on companies, marking an era of lifetime active participation. Accidents due to falls are the most common workplace accidents; since 2015, the Ministry of Health, Labour and Welfare has been implementing the “STOP! Fall Accident Project.” However, the number of fatalities and injuries resulting in more than 4 days of absence increased by 18.9% in the fiscal year 2023, with falls being the most prevalent (34 000 people) compared with the fiscal year 2017 (Laborer Injury and Illness Report). The increasing proportion of older adult workers necessitates the urgent development of fall detection and prevention systems.

Various fall risk assessment tools for older adults have been developed in hospitals^1^^,^^2^ and in community settings.^3^ The fall risk assessment tools currently used for the elderly do not show sufficiently high predictive validity for differentiating between high and low fall risk.^4^ A fall prevention system attempts to predict and reduce the risk of falls. The latest research trends in fall detection and prevention systems use machine learning (ML) algorithms in inpatient and community-dwelling older adults.^5^ It is noted that the accuracy is limited owing to the origin of the data being electronic medical and nursing records.^6^ Recent studies on fall risk prevention have used wearable sensor-based technologies for fall risk assessment,^7^ but video-based fall risk prediction systems have not been developed.

Considering the increasing incidence of workplace fall accidents, walking in the workplace involves frequent initiation and cessation of walking rather than simple linear walking. It has been observed that compromised balance control during walking initiation can lead to falls.^8^ Gait analysis is extensively used for medical diagnosis, rehabilitation, and biometric identification. Clinical gait analysis is often conducted in a gait lab employing a 3D motion capture system and a pressure-sensing walkway. Gait assessment can be conducted outside a gait laboratory if the subject uses wearable inertial sensors.^9^

Abnormalities in the first 3 steps of gait initiation have been observed in patients with Parkinson disease with freezing of gait.^10^ However, motion capture suits used in laboratory experiments are expensive and not applicable in the workplace. Using wearable sensors in real-world settings presents significant challenges, whereas video-based approaches offer greater versatility and hold more substantial value. Previous research has predominantly focused on assessing the relationship between walking speed and fall risk. Although walking speed serves as a reliable indicator of physical fitness and strength in older adults, measuring it necessitates a 10-m space, which may not be practical in workplace environments. When investigating the circumstances of falls in the workplace, gait initiation was often cited, leading us to concentrate on the first 3 steps.

Therefore, this study aimed to develop and validate a high fall risk prediction model using ML and video-based first 3 steps for middle-aged workers.

## 2. Methods

### 2.1. Study design

A fall prediction model was developed using an ML-based algorithm in a cross-sectional study design. ML refers to a technology that enables computers to learn from data and improve their performance based on experience. Specifically, ML uses algorithms and statistical models to recognize patterns, classify data, and make predictions based on input data. Supervised learning is a type of ML where an algorithm is trained on a labeled dataset, meaning that each training example includes both the input data and the corresponding correct output (label). The goal of supervised learning is to learn a mapping from inputs to outputs that can be used to make predictions on new, unseen data. For example, applications of supervised learning include medical diagnosis and image recognition.

### 2.2. Participants

For this study we enrolled 192 participants (field experiments and training data) and 28 participants (laboratory experiments and validation data). The inclusion criteria were individuals aged 40-69 years, whereas the exclusion criteria comprised individuals having trouble in walking. The participants for the training data were recruited from hospital staff and local residents living near hospitals, whereas the participants for the validation data were recruited from a high-fall-risk population through a clinical trial panel. Data collection included occupational falls, history of falls during the past year,^11^^,^^12^ medication, and blood pressure categories. “Occupational fall” was defined as a fall occurring at work that results in a work-related injury requiring an absence from work of 4 or more days. “Fall in the past year” was defined as any fall that occurred within the past year, irrespective of whether it took place during work or outside of work. The study protocol was approved by the institutional review board of Kyoto Medical Center (No. 21-057), and the protocol of the study was registered at the University Hospital Medical Information Network Center (UMIN000051453).

### 2.3. Gait analysis

**Figure 1:**
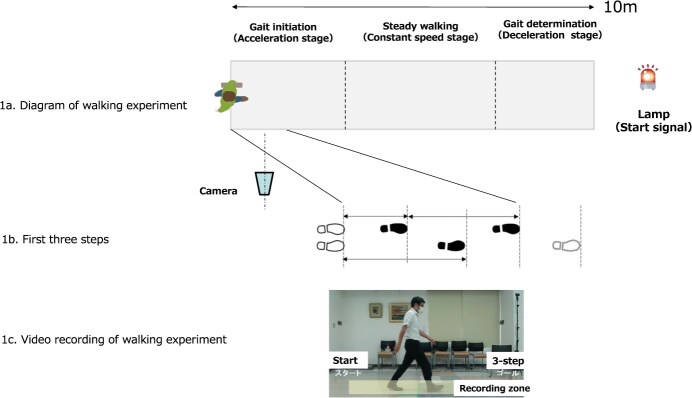
Gait analysis during the first 3 steps.

The walking distance was 10 m, and the participants were asked to walk normally 3 times. They were instructed, “When the lamp ahead lights up, walk towards the goal as you usually do.” We used an RGB (red, green, blue) camera to capture the gait characteristics during the first 3 steps (Figure 1).

### 2.4. Definition of joint points and coordinate systems

**Figure 2:**
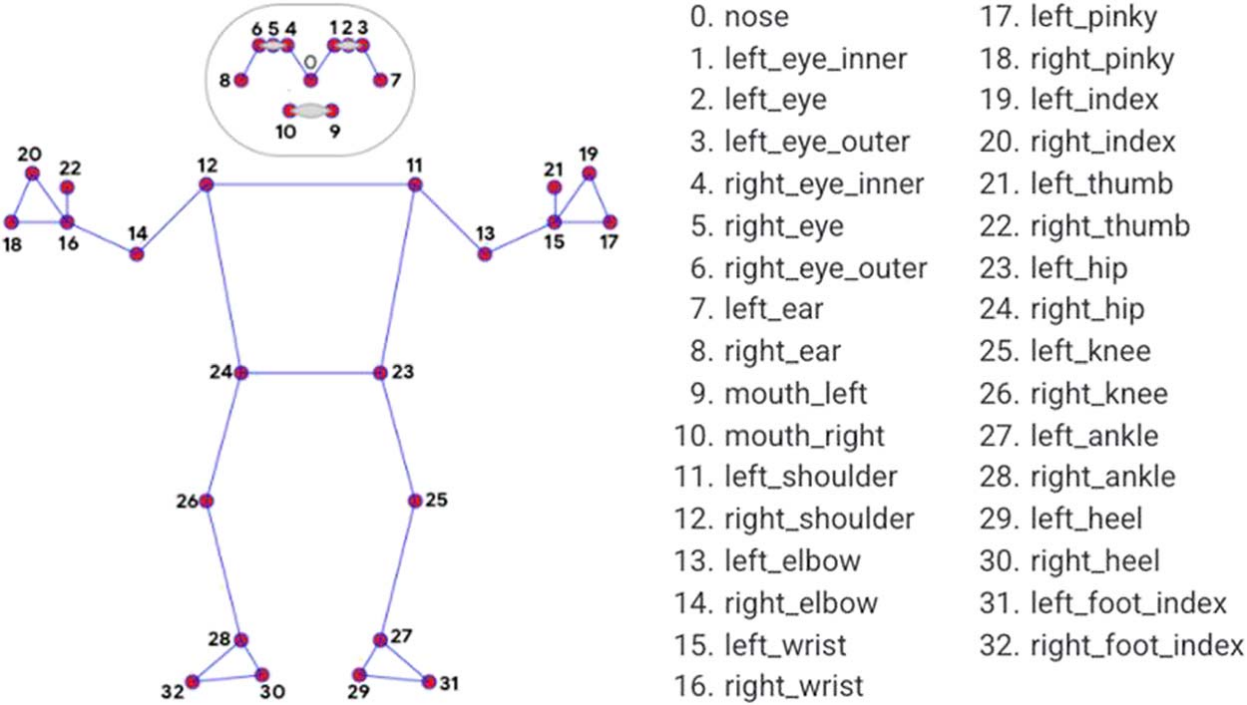
The definition of joint points in the MediaPipe Pose.

We used MediaPipe Pose for skeleton estimation and extracted skeleton coordinates from images.^13^ To extract features based on body parts, the definition of each joint point was based on the definition of the joint points in the MediaPipe Pose. Figure 2 illustrates the definition of joint points in MediaPipe Pose, and a Figure 2 shows the joint points used based on these definitions. Some joint points were not directly available in the MediaPipe Pose, so they were calculated as the midpoints or centroids of the extracted joint points. The coordinate system was defined as a right-handed coordinate system with the vertical direction as the y-axis and the forward direction as the z-axis. Iturrieta et al^14^ reported that, using the Bland-Altman method, MediaPipe® and a webcam-based sensor for the upper limb can be used interchangeably with a goniometer. Additionally, the MediaPipe algorithm demonstrated high performance in tracking lower limb movements.^15^

### 2.5. Definition and segmentation of the gait cycle

**Figure 3:**
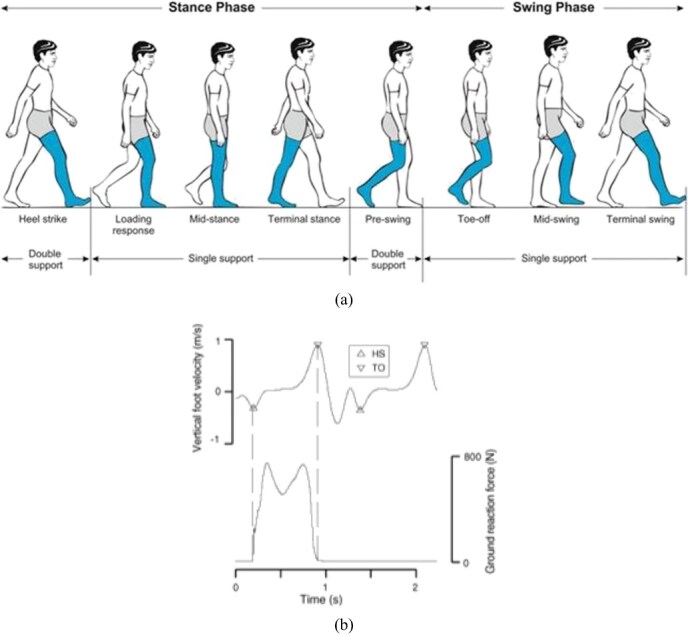
The definition of gait cycle and the extraction of the data.

Regarding the gait cycle, as illustrated in the Figure 3, 1 gait cycle is defined from the moment 1 foot makes contact with the ground until the same foot on the same side makes contact again (Figure 3). Velocity data from the vertical axis of the ankle joint were used to segment each step. The velocity data were obtained by differentiating the time-series data of the vertical axis of the ankle joint. Following prior literature,^16^^,^^17^ the extraction of gait cycles was conducted by identifying the local minima of velocity data as the “moment when the heel contacts the ground.” The gait features calculated for each gait cycle were aggregated using the following method. Specifically, aggregation is performed based on the left–right difference between the first step, second step, third step, and the difference between the first and second steps.

**Figure 4:**
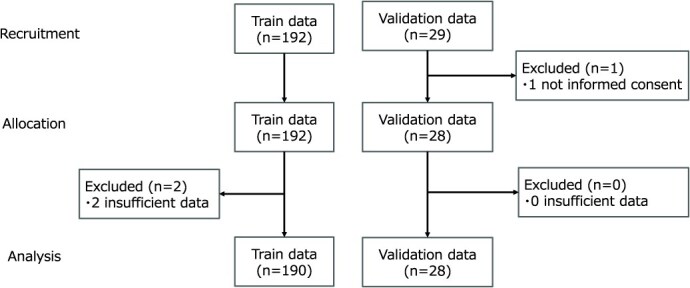
Flow diagram of the recruitment, allocation, and analysis.

Two types of joint angles were calculated: (1) internal joint angles—angles calculated from 3 points on a body part, such as the knee joint; and (2) external joint angles—relative angles in the global coordinate system of vectors consisting of 2 points on a body part during the gait cycle.

To calculate the joint displacement features, we set the gait direction described at the beginning of each section within 1 gait cycle as the axis of the world coordinate system and computed the time-history waveforms. For the vertical axis of the time-history waveform, we corresponded to the x, y, and z axes of the world coordinate system, respectively. We then calculated the basic statistical parameters for these time-history waveforms and aggregated them to define joint displacement features. This resulted in the definition of 12 joint displacement features. Based on the definition of 1 gait cycle described previously, which is based on the segmentation of 1 step, we defined a gait cycle and established 7 temporal features related to the swing, stance, and double support phases within the gait cycle.

In this study, the calculation of gait speed was based on the movement along the z-axis of the global coordinate system of the swing foot. Below are examples of gait features calculated within 1 gait cycle: walking speed and movement along the z-axis of the swing foot within 1 gait cycle divided by the duration of the gait cycle. Maximum swing phase velocity: as defined as maximum velocity of the swing foot during the swing phase within 1 gait cycle.

Considering the human body as a rigid link model, the velocity and joint displacement features vary depending on the height. In this study, we divided the joint displacement features by the height. Additionally, for the velocity features, we divided by the square root of the height.

There are 77 gait features, excluding step length at the first step. These features are categorized as follows: Speed & Acceleration (3 features), Time (5 features), Distance & Height (7 features), and Angles (11 features), measured during the first, second, and third steps (see Appendix).

### 2.6. Selection of key gait features for analysis

In the first stage, each extracted gait feature was evaluated for its ability to predict fall history using receiver operating characteristic–area under the curve (ROC-AUC), and about 10 features were selected. In the second stage, to maintain the interpretability of the estimation model, a comprehensive search was conducted for combinations of features ranging from 3 to 5. Generally, for handling imbalanced datasets with small sample sizes, techniques such as synthetic minority oversampling for oversampling the minority class are considered. However, since the dataset was limited to about 100 samples for each gender, with a positive-to-negative ratio of 1:9 in the training data, the undersampling method NearMiss was applied instead. Additionally, a stratified group *k*-fold cross-validation method was used for the combination search. To ensure robust validation, the number of folds was set between 3 and 5, and the seed values were changed twice for each fold. The model was determined based on the average evaluation metrics obtained for each combination of fold numbers and seed values. The ratio of positive (fall history) to negative (no fall history) cases was tested at 5 levels: 1:5, 1:4, 1:3, 1:2.5, and 1:1. Based on the results, a positive-to-negative ratio of 1:4 was found to be optimal for this dataset, and the estimation model was developed using the near-miss sampling method^18^ with this ratio.

### 2.7. Sample size

The sample size in the training data was calculated using the R package *pmsampsize* with the following parameters: C-statistic of 0.8, 3 parameters, and a prevalence rate of 0.2, which resulted in a requirement of 134 participants. *k*-Fold cross-validation is a widely used technique for model evaluation, particularly beneficial for small sample sizes. However, there is no definitive guideline regarding the minimum sample size required for effective implementation. Feature selection was performed following a simulation study based on a real dataset by Peduzzi et al,^19^ which concluded that 5-10 events per predictor variable are necessary. Based on this criterion, we selected 3 to 5 gait features.

### 2.8. Statistical analysis

Data analysis was performed using Python and R. Descriptive data were reported as frequency and percentages. Continuous data with a normal distribution were presented as mean and SD. Subsequently, we constructed a fall prediction model using logistic regression based on a combination of these 3 gait characteristics. Model evaluation was conducted through cross-validation, assessing model performance using the AUC. Data are presented as AUC with 95% CIs. In cases of imbalanced data, it has been reported that the precision-recall (PR) curve provides a more suitable evaluation for datasets with fewer positive samples compared with the ROC curve.^20^ In this study, we optimized the predictive model using the area under the PR curve (PR-AUC) as the evaluation metric. The ROC-AUC is only inflated by an imbalance in simulations where changing the imbalance changes the score distribution. By contrast, the PR-AUC changes drastically with class imbalance.^21^ An AUC greater than 0.9 was considered as excellent, 0.8-0.9 as very good, 0.7-0.8 as good, 0.6-0.7 as sufficient, 0.5-0.6 as bad, and lower than 0.5 as test not useful.^22^ Participants with missing data were excluded from the analysis.

## 3. Results

### 3.1. Characteristics of the participants

As shown in Figure 1, 190 training data and 28 validation data were analyzed. Two participants were excluded from the analysis due to a lack of coordination, with the hands and feet moving on the same side (Figure 4). The mean (SD) age and body mass index (BMI) of the training data (*n* = 190, men 48.9%) were 54.5 (7.7) years and 22.5 (3.2) kg/m^2^, respectively. For the validation data (*n* = 28, men 53.6%), the mean (SD) age and BMI were 52.3 (6.0) years and 23.1 (2.8) kg/m^2^, respectively. In the training data, the prevalence of accidental falls and history of falls was 3.2% and 12.0%, respectively. In contrast, in the validation data, the prevalence of accidental falls and history of falls was 3.6% and 25.0%, respectively (Table 1). There was no difference in the rate of history of falls during the past year between men and women in training data (*P* = .377) or validation data (*P* > .999).

**Table 1:** Characteristics of the study participants.^a^

**Variables**	**Training data** **(*n* = 190)**	**Validation data** **(*n* = 28)**
**Age, y**	54.5 (7.7)	52.3 (6.0)
**Men, %**	48.9	53.6
**Body mass index, kg/m** ^**2**^	22.5 (3.2)	23.1(2.8)
**Occupation, %** **Agriculture, forestry, and fisheries** **Labor** **Sales and services** **Administration and security** **Others**	1.1 6.8 42.1 25.3 24.7	0 28.6 25.0 21.4 25.0
**Accidental fall, %** **History of fall during the past year** **All** **Men** **Women**	3.2 12.1 9.7 14.4	3.6 25.0 26.7 23.1

a Data are mean (SD) or percentage.

### 3.2. Gait features

In men, we extracted 13 gait features (3, 4, and 6 in the first, second, and third step, respectively) linked to a history of falls in the past year. Similarly, in women, we found 10 gait features (3, 3, and 4 in the first, second, and third step, respectively) associated with a history of falls in the past year (Table 2). The reproducibility (eg, walking speed during first step) was 0.627 (95% CI, 0.522-0.713; *P* < .001).

**Table 2:** Gait features in men and women associated with history of fall during the past year.

**Step**	**Men**	**ROC-AUC (95% CI)**	**Women**	**ROC-AUC (95%CI)**
**1st step**	Head sway Swing time Swing phase ratio	0.520 (0.322-0.717) 0.660 (0.453-0.867) 0.647 (0.436-0.858)	Swing time Swing phase ratio Hip ROM	0.440 (0.268-0.612) 0.606 (0.435-0.777) 0.466 (0.293-0.640)
**2nd step**	Hip ROM Swing time Knee ROM Head angle	0.533 (0.293-0.773) 0.652 (0.426-0.879) 0.611 (0.365-0.858) 0.595 (0.419-0.771)	Body sway Head angle ROM of the knee joint	0.659 (0.517-0.801) 0.616 (0.446-0.787) 0.627 (0.492-0.761)
**3rd step**	Maximum heel height Swing time Head sway Head angle Stride Knee ROM	0.737 (0.560-0.913) 0.625 (0.428-0.822) 0.676 (0.498-0.854) 0.593 (0.420-0.765) 0.541 (0.299-0.783) 0.743 (0.590-0.897)	Knee ROM Body sway Head angle Stance time	0.565 (0.391-0.738) 0.525 (0.356-0.694) 0.612 (0.437-0.787) 0.552 (0.392-0.713)

Abbreviations: AUC, area under the curve; ROC, receiver operating characteristic; ROM, range of motion.

### 3.3. ROC-AUC analysis

In men, of the 13 gait features, we identified 3 with an AUC of 0.909 (95% CI, 0.879-0.939), indicating excellent performance. Conversely, in women, among the 10 gait features, we identified 5 with an AUC of 0.670 (95% CI, 0.621-0.719), indicating sufficient performance (Table 3).

**Table 3:** ROC-AUC and PR-AUC analysis according to the sex category.

**Sex**	**Gait features**	**Training data**	**Validation data**
**Men**	1st step: Swing phase ratio 3rd step: Knee ROM Maximum heel height	ROC-AUC: 0.702 (95% CI: 0.694-0.710) PR-AUC: 0.210 (95%CI: 0.204-0.216)	ROC-AUC: 0.909 (95% CI: 0.879-0.939) PR-AUC: 0.780 (95%CI: 0.740-0.820)
**Women**	1st step: Hip ROM 2nd step: Vertical trunk movement Knee ROM 3rd step: Knee ROM Trunk sway	ROC-AUC: 0.630 (95% CI: 0.622-0.638) PR-AUC: 0.280 (95%CI: 0.274-0.286)	ROC-AUC: 0.670 (95% CI: 0.621-0.719) PR-AUC: 0.630 (95%CI: 0.576-0.684)

Abbreviations: AUC, area under the curve; PR, precision recall; ROC, receiver operating characteristic; ROM, range of motion.

## 4. Discussion

To the best of our knowledge, this is the first study to develop and validate a fall risk prevention system for middle-aged workers using video-based gait analysis data. Of the 77 gait features extracted from the first 3 steps, we identified 3 with excellent performance in men and 5 with sufficient performance in women.

### 4.1. First 3 steps and fall risk

In this study, we specifically examined the initial 3 steps in a middle-aged population. Abnormalities in the first 3 steps of gait initiation were commonly observed in patients with Parkinson disease who experience freezing of gait. In both men and women, the gait characteristic associated with predicting falls was the range of motion (ROM) of knee flexion in the third step. However, the relationship between knee ROM and fall risk in the middle-aged population remains unclear. Nonetheless, decreased passive lower extremity ROM and flexibility are recognized contributors to falls in older adults.^23^ In men, the maximum heel height was associated with an increased risk of falls. Tripping is a significant cause of falls among older adults. Consequently, foot clearance, which refers to the height of the foot above the ground during the swing phase of gait, may be a crucial factor in comprehending the intricate interplay between gait patterns and falls.^24^ Older adults with reduced physical function and signs of frailty mainly display a longer duration of gait initiation and decreased first step length than non-frail older adults.^25^ In this study, the swing phase ratio was found to be correlated with falls in men. The most significant gait parameters included walking speed, maximum knee flexion angle, stride length, and the stance-swing phase ratio, which served to quantify the overall gait quality.^26^ In the women in this study, falls were associated with the hip ROM or body sway. Hip ROM is specifically linked to Parkinsonian gait.^27^ Additionally, trunk kinematics have been correlated with fall risk in older adults.^28^ For both trips and slips, the ability to limit trunk motion has consistently discriminated older adults who fall from younger and older adults who can avoid falling.^29^ In the middle-aged population, these combinations of gait characteristics may affect the outcomes.

Why the accuracy of the validation data was higher than the training data is unclear. Several potential reasons were speculated: overfitting in the training data, differences in data distribution, and issues with data preprocessing or splitting. Further investigation, including biomechanical analysis, is necessary to confirm these findings.

### 4.2. Sex differences in the accuracy for fall risk

The reason for sex differences in the accuracy of fall risk is unclear. The gait analysis data revealed significant sex-based differences in this study. Women demonstrated greater pelvic obliquity compared with men while walking, but they also showed a more stable torso and head. Additionally, women exhibited increased rotation of the pelvis and torso in the transverse plane, along with a more pronounced arm swing in this study. Sex-based differences were observed in the gait patterns of 98 healthy Korean adults (47 women and 51 men). Women were shorter in height and leg length than men. Despite this, their cadences and pelvic widths were comparable to those of men. However, women walked at a slower pace because of their shorter stride lengths. Additionally, women exhibited even shorter stride lengths and narrower step widths but walked at a pace similar to that of men in this study. Female participants walked with their pelvis tilted more anteriorly and demonstrated more up-and-down oblique motion, with the hip joints being more flexed, adducted, and internally rotated, and knee joints exhibiting more valgus angles.^30^ These differences are attributed to anatomical and habitual sex-related features of gait. Structural differences between the sexes are often implicated as a factor in this discrepancy.^31^

It is possible that factors other than gait analysis data may be associated with falls in women compared with men. Several factors could contribute to this difference, including: biomechanical differences, hormonal factors, footwear choices, age-related changes, and psychosocial factors. Changes in hormone levels, particularly during menopause, can impact bone density, muscle strength, and balance, potentially increasing fall risk in women. Women may be more likely to wear footwear, such as high heels, that can compromise stability and increase fall risk. Age-related muscle loss (sarcopenia) and joint issues may affect men and women differently, influencing fall risk. Psychosocial factors, such as fear of falling and differences in physical activity levels, may also vary between men and women, contributing to different fall risks. Considering these factors alongside gait analysis data may provide a more comprehensive understanding of fall risk in women compared with men.

### 4.3. Fall risk assessment in the workplace

Gait is also affected by age.^32^ According to a retrospective study involving 1164 older workers aged ≥60 years, assessing medication use, functional strength, standing balance, and visuospatial ability during regular health checks in the workplace may be beneficial in identifying older workers at risk of occupational falls.^33^ Clinical tests and questionnaires have shown limited diagnostic value in assessing fall risk in older adults.^34^ Traditional fall risk assessments may not be suitable for older adults with cognitive impairment because of their reliance on attention and recall. Therefore, there is growing interest in using objective technology-based fall risk assessment tools to evaluate falls in this population.^35^ Recently, fall risk assessment in older adults has been conducted using ML, lumbar sensors, accelerometers, and wearable sensors during timed up-and-go (TUG) tests.^36^ However, no studies have been conducted on video-based gait analysis of workers. Not all individuals can wear wearable devices, particularly in circumstances where they do not own a smartphone or are in environments that prohibit their use. Furthermore, video-based gait analyzers are capable of recording individuals in their natural state and can be strategically placed in high-risk areas to identify individuals at risk of falling early on. This approach is also more cost-effective and less labor-intensive, as it eliminates the need to distribute wearable devices to all individuals. In the future, we aim to exclude unreliable data by employing a data-driven deep neural algorithm to detect deceptive walking behavior.

### 4.4. Strengths and limitations

The strength of this study is that it included a noninvasive video-based gait analyzer and did not include a wearable sensor. This system is convenient, versatile, and has various practical applications. However, this study had several limitations, including the use of self-reported questionnaires. We did not obtain specific numbers of falls, and anyone who had fallen was defined as having experienced a fall incident, with the frequency of falls categorized as either no fall or 1 or more falls. Additionally, because the participants were between 40 and 69 years of age, the generalizability of the findings is limited. Additionally, our study did not report severe obesity. The obese group exhibited slower walking speed and shorter stride length than the nonobese group. Additionally, they spent more time in the stance phase and on double support during walking. Greater hip adduction was observed in the obese group during the terminal stance and pre-swing phases. Significantly higher maximum knee adduction angles were noted in the obese group during both stance and swing phases. Furthermore, the obese group showed significantly greater ankle eversion angles from the mid-stance to the pre-swing phase. Moreover, there was a reduction in peak ankle plantar flexor moment and an increase in ankle inversion moment in the severely obese group.^37^ Other sex-related differences in gait have been observed in patients with knee osteoarthritis.^38^ The recruitment for the training data focused on hospital staff and local residents living near hospitals, whereas the validation data were collected from a high fall-risk population recruited through a clinical trial panel. In this study, the generalizability was limited because certain factors were excluded. There are several definitions of “fall.” The Prevention of Falls Network Europe provides the gold standard definition as “an unexpected event in which the participant comes to rest on the ground, floor, or lower level.”^39^ In a meta-analysis by Welsh et al,^40^ 32% of studies used this definition, whereas 45% did not explicitly define falls, and the remaining studies used definitions adapted from the gold standard. Similarly, a meta-analysis by Hauer et al^41^ found that, among 90 publications, 44 provided no definition of “fall.” For our research in real-world workplace settings, we used definitions adapted from the gold standard; however, further examination—including the gold standard definition—is needed to address these issues in the future.

### 4.5. Clinical implications

Falls are a leading cause of injury, decreased work productivity, and fatalities in the workplace.^42^ Muscle strength and balance are significant modifiable factors contributing to falls in older adults^43^ but not in middle-aged people.^44^ Therefore, fall prevention measures are necessary for the middle-aged population. Recent advances in mobile technologies provide opportunities to integrate contextual information about patient behavior and the environment with physiological health data to predict falls.^45^ However, not all individuals can wear wearable devices, particularly in circumstances where they do not own a smartphone or are in environments that prohibit their use in the workplace. Furthermore, video-based gait analyzers are capable of recording individuals in their natural state and can be strategically placed in high-risk areas to identify individuals at risk of falling early on. This approach is also more cost-effective and less labor intensive, as it eliminates the need to distribute wearable devices to all individuals. The social implementability of this system is deemed high; however, challenges remain regarding the optimal locations for installation (eg, entrances, stairs, slippery areas) and the methods of providing feedback to individuals at high risk of falling. However, the accuracy of these models was not satisfactory in women. It was observed that gait was influenced by factors such as obesity and alcohol consumption.^46^ For women, further development of prediction models that consider various conditions is necessary to improve accuracy, especially in middle-aged working populations.

## 5. Conclusion

These findings suggest that fall risk prediction can be developed based on ML and the first 3 steps in men; however, the accuracy was only “sufficient” in women. Further development of the formula for women is required to improve its accuracy in the middle-aged working population.

## Supplementary Material

Web_Material_uiae075

## Data Availability

The data that support the findings of this study are available from the corresponding author, upon reasonable request.
