# Supplementary material for: Application of machine learning for detecting high fall risk in middle-aged workers using video-based analysis of the first 3 steps
Source: J Occup Health. 2025 Jan 10;67(1):uiae075. doi: 10.1093/joccuh/uiae075 (PMC11848130; doi:10.1093/joccuh/uiae075)
Supplement: Web_Material_uiae075 [file web_material_uiae075.zip › 241113Supporting information.pdf]

Appendix. Gait features in the first three steps.

Categories	Gait features
Speed & Acceleration (3 features)	Walking speed, Maximum swing leg speed, Maximum acceleration per step
Time (5 features)	Gait cycle, Stance time, Stance phase ratio, Swing time, Swing phase ratio
Distance & Height (7 features)	Step length, Stride, Maximum heel height, Vertical movement of the chest, Waist vertical movement amplitude, Wrist forward-backward swing amplitude, Knee vertical swing amplitude
Angles (11 features)	Heel strike (pitch angle), Toe-off angle, Head angle, Head sway, Elbow swing angle, Trunk sway, Vertical trunk movement, Hip ROM, Knee ROM, Ankle ROM, Maximum angular velocity during swing phase
